# Investigating the relationship between gut microbiota and electrocortical signatures of feedback processing: an ERP study

**DOI:** 10.1007/s00213-025-06878-9

**Published:** 2025-08-29

**Authors:** Sabrina Lenzoni, Kirsty Hunter, Nadja Heym, Bryony Heasman, Stephanie Blanco, Gemma Walton, Glenn Gibson, Carlos Poveda, Thomas Baguley, Grace Y. Wang, Daniel C. Mograbi, Alexander Sumich

**Affiliations:** 1 Department of Psychology, Pontifical University of Rio de Janeiro, Rio de Janeiro, 22451-900 Brazil; 2 Department of Psychology, Nottingham Trent University, Nottingham, NG1 4FQ UK; 3 Department of Sport Science, Nottingham Trent University, Nottingham, NG1 4FQ UK; 4 Department of Food and Nutritional Sciences, University of Reading, Reading, RG6 6AH UK; 5 School of Psychology and Wellbeing, University of Southern Queensland, Toowoomba, 4300 Australia; 6 Institute of Psychiatry, Psychology & Neuroscience, King’s College London, London, SE58AF UK; 7 Department of Psychology, Auckland University of Technology, Auckland, 1010 New Zealand

**Keywords:** Feedback-related negativity, Gut microbiota, ERPs, EEG

## Abstract

**Rationale:**

Evaluative processing of action outcome is considered crucial for learning and adaptive adjustments of behaviour. Feedback-related negativity (FRN) is an event-related potential elicited by feedback presentation, with implicated neural sources in the anterior cingulate cortex. Bidirectional communications within the brain-gut-microbiota axis modulate cognition and behaviour, and microbial composition has been associated with medial prefrontal cortex function and clinical risk for depression.

**Objectives:**

The present study aimed to investigate associations between specific gut microbiota and the FRN.

**Methods:**

Twenty-nine healthy participants completed self-report measures of depression and a Faces and Feedback task during electroencephalography recording. Select implicated microbiota genera were enumerated from stool samples (*Clostridium*, *Lactobacillus)*, along with plasma C-reactive protein (CRP) as an index of systemic inflammation.

**Results:**

FRN amplitude for positive feedback was positively correlated with microbiota abundance. The relationship between *Clostridium* and FRN was confirmed by multilevel modelling analysis, controlling for depression and CRP. The latter was positively associated with FRN amplitude.

**Conclusions:**

Findings suggest that the brain-gut-microbiota-axis may modulate or be modulated by self-monitoring processes. The current work provides insights into neurophysiological mechanisms underlying reward processing and indicates novel directions for therapeutic interventions, such as those that modulate the gut microbiome.

**Supplementary Information:**

The online version contains supplementary material available at 10.1007/s00213-025-06878-9.

## Introduction

The brain-gut-microbiota axis (BGMA; Kelly et al. 2016) refers to the multi-directional interactions between the gut and the brain (Grenham et al. 2011; Carabotti et al. 2015). These communication pathways comprise the central nervous system (CNS), autonomic nervous system (ANS), enteric nervous system, neuroendocrine and neuroimmune systems, and the intestinal microbiota (i.e., the community of micro-organisms which reside in the gut; Carabotti et al. 2015; Cryan and Dinan 2012; Mayer 2011). Brain-to-gut pathways are involved in homoeostatic regulation through hierarchical networks from physiological responses (e.g., intestine, gastric, and enteric reflexes) to top-down loops involving limbic structures (hypothalamus, amygdala, insula, anterior cingulate cortex; Mayer 2011). On the one hand, top-down control is recruited in response to environmental factors, such as stressors, and serves as integration of interoceptive and exteroceptive signals to modulate intestinal homeostatic regulation (Mayer 2011; Cryan et al. 2019). On the other hand, gut microbiota contributes to regulation of CNS and behaviour, in a bottom-up fashion through direct (i.e. and via vagus nerve; Bonaz et al. 2018; Bravo et al. 2011) and indirect (immune and endocrine; Sudo et al. 2004) systems, leading to altered neuroimmune signalling (Erny et al. 2015; Cryan and Dinan 2015), stress responsiveness, and neurotransmission (e.g., O’Mahony et al. 2015).

Functional Magnetic Resonance Imaging (fMRI) studies have shown that the association between alterations in the gut microbiota population and reduced default mode network connectivity in end-stage renal disease patients was mediated by systemic inflammation (Wang et al. 2019). Callaghan et al. (2020) reported that bacterial levels were correlated with medial and lateral PFC, posterior cingulate cortex, and precuneus task-related activity in children exposed to adversity. Moreover, in healthy participants, microbial diversity was associated with executive control network connectivity, interconnectivity between executive control, default mode and sensorimotor networks (Cai et al. 2021) and insular connectivity (Curtis et al. 2019). Taken together, these studies suggest an association between gut microbiota composition and activation of neural networks recruited in performance monitoring processes.

Self-monitoring is essential to perform successful goal-directed behaviours, evaluate the action outcomes and adjust behaviours, by learning how to avoid mistakes (Ullsperger et al. 2014). Electroencephalography (EEG) research identified event-related potential (ERP) markers of performance monitoring, the medial frontal negativities (MFN). One type of MFN, the Feedback-Related Negativity (FRN; Miltner et al. 1997) is thought to reflect performance feedback evaluation and reward processing (Hajcak et al. 2006, 2007). The FRN is a negative deflection peaking at around 250–300 ms following feedback presentation and has higher amplitude at mid frontal electrode sites in response to negative compared to positive feedback (Krigolson 2018). The primary neural source of FRN has been localized in the anterior cingulate cortex (ACC; Bellebaum and Daum 2008; Gehring and Willoughby 2002; Nieuwenhuis et al. 2005; Zhou et al. 2010), posterior cingulate (Badgaiyan and Posner 1998; Luu et al. 2003; Hayden et al. 2008) and basal ganglia (Martin et al. 2009; Carlson et al. 2011; Foti et al. 2011). According to the reinforcement learning theory (RL-T; Holroyd and Coles 2002; Holroyd and Yeung 2012), the FRN reflects a learning prediction error, that is computed by the basal ganglia, as a mismatch between outcome and expectations, and signalled through dopaminergic phasic activity to the ACC to promote performance adjustments (for a discussion of different theoretical accounts see Walsh and Anderson 2012). Previous studies showed that changes in ANS reactivity, mostly indexed by heart rate and skin conductance responses, were associated with error commission and negative feedback, suggesting that ANS activity can trigger adaptive preparation mechanisms for behavioural adjustments (Ullsperger et al. 2014). However, limited ERP research has explored the contribution of peripheral signals in relation to feedback and reward processing. Specifically, Kimura (2019) investigated the effect of cardiac cycle on feedback-related ERPs and reported that systolic activity modulated the FRN for positive (gain), but not negative (loss) feedback, suggesting that afferent peripheral signals contribute to reward processing. Furthermore, authors investigating MFNs in relation to psychopathology and neurodegeneration (Walsh and Anderson 2012; Tobias and Ito 2021; Bellato et al. 2021; Lenzoni et al. 2022) have proposed that blunted MFN may be a biomarker of depression (Proudfit 2015; Brush et al. 2018; Clayson et al. 2020).

A growing body of evidence indicates that gut microbiota plays a role in modulating dopaminergic signalling, which subserve reward processing pathways. Animal research has shown that alteration of microbial composition triggers changes in dopaminergic neurotransmission and underlying mesocorticolimbic circuits (Hamamah et al. 2022). It has been suggested that intestinal microbial composition may affect dopaminergic receptor expression. However, the direct and indirect effects of microbial changes on neurotransmission is still poorly understood (González-Arancibia et al. 2019). As recently reviewed by (García-Cabrerizo et al. 2021), gut microbiota may be involved in modulating the reward system and regulate food, social, sexual and drug reward processes. Microbial numbers in the human gastrointestinal tract range approximately between 10^13^ and 10^14^ cells/g, with highest abundance in the colon. The two most prominent phyla are Firmicutes (including genera such as *Lactobacillus* and *Clostridium)* and Bacteroidetes, together accounting for around ¾ of the microbiota numbers (Eckburg et al. 2005; Rinninella et al. 2019). The relationship between gut microbes and brain health and function is an emerging area of research with considerable interest being focused on *Lactobacillus* and *Clostridium* genera. Supplementation with probiotic (i.e., introducing the microbiota through diet) *Lactobacillus* for example, has been shown to improve depression- and anxiety-like behaviour and neurocognitive factors in animal models (Li et al. 2018; Liang et al. 2015; Sun et al. 2018, 2020, 2021a, 2021b; Zhang et al. 2019). In humans, gut colonization of *Clostridium* has been proposed to be involved in autism spectrum disorder pathogenesis and symptomatology (Alshammari et al. 2020; Kandeel et al. 2020). Furthermore, in an exploration of the relationship between *Clostridium* and stress, a rise in *C. perfringens* was observed in students throughout the final examination period (Mullié et al. 2002); however, the study did not include any direct measure of mood. Further studies showed that probiotic supplementation could ameliorate major depression disorder symptoms (*L. acidophlus*,* L. casei* and *Bifidobacterium bifidum* (Akkasheh et al. 2016), post-natal anxiety depression (*L. rhamnosus;* Slykerman et al. 2017) and anxiety symptoms (but not depression) in chronic fatigue syndrome (*L. casei*; Rao et al. 2009). However, other studies reported null effects of prebiotics (i.e., non-digestible nutrients degraded by microbiota to lead to health benefits) with *Lactobacillus* on depressive symptoms (L. helveticus; Romijn et al. 2017; Romijn and Rucklidge 2015) or stress-related measures (Kelly et al. 2017). Considering the range of probiotic strains, differences in sample characteristics (such as clinical group, age, sample size etc.), and the heterogeneous choice of self-report mood measures, a more complex interaction between microbiota and (neuro)psychological outcomes can be hypothesized. Interestingly, Heym et al. (2019) reported that faecal abundance of *Lactobacillus* spp. was indirectly related to cognitive depression, but directly related to self-judgment, suggesting that microbiota composition may be related to self-referential mechanisms. This is in line with Steenbergen et al. (2015) who found a strong effect of multispecies probiotic supplementation for rumination-type symptoms.

Critically, limited EEG research has focused on the association between the gut microbiome and neurocognitive functioning. Following 4-week supplementation with *Bifidobacterium longum* 1714 (1 × 10^9^ colony-forming units per day) in healthy adults, resting state Fz mobility was higher following active treatment compared to baseline and placebo conditions, and Cz theta power was lower following active treatment as compared to placebo, with no effect on oddball P300 (Allen et al. 2016). Kelly et al. (2017) reported that after 4-weeks *L. rhamnosus* supplementation (1 × 10^9^ colony-forming units per day) in healthy males, the only difference between placebo and probiotic conditions was in F3 zero-crossing, but no effect for other EEG measures. Moreover, Canipe et al. (2021) showed that, in older adults, higher microbial diversity was associated with larger frontal N1 amplitude for targets in a detection task and, with shorter frontal N2 latency and larger temporal P3 amplitude for a *familiar* condition during an oddball task. Follow-up analyses showed that these measures had specific associations with different phyla. N1 was positively associated with Proteobacteria, Tenericutes and Cyanobacteria, shorter N2 latency was associated with Cyanobacteria, and P3 was negatively associated with Proteobacteria. Taken together, the evidence suggests the existence of specific relationships between neurophysiological measures and the gut microbiota.

The aim of the current study is to explore the relationship between lower gut microbiota composition and neurophysiological correlates of reward processing. Specifically, this study will focus on *Lactobacillus - Enterococcus* and *Clostridium histolyticum* group abundance and association with FRN. To the best of our knowledge, this is the first study exploring the association between gut microbiome and performance monitoring ERPs. We hypothesize that, after accounting for direct effects of inflammation and depression, higher microbial abundance would be associated with larger FRN amplitude, thus reflecting a positive association between gut signals and enhanced performance monitoring processes. Confirmation of such as relationship would offer a novel neurocognitive target for interventions affecting BGMA.

## Methods

### Participants

Twenty-nine healthy adults were recruited at Nottingham Trent University. Participants were included in the study if they were aged over 18 and fluent in English. Exclusion criteria were history of neurological, psychiatric, renal, cardiovascular, or hepatic diseases; alcohol misuse; antibiotic use (within the previous 3 months); regular intake of anti-inflammatory and prebiotic/probiotic/synbiotic (within the previous month); pregnancy. Two participants were breastfeeding, 9 participants reported use of dietary supplements.

An online survey was used to collect demographic data and assess depression. Participants who were eligible were invited to the university campus, where they provided blood and stool samples, and performed a computerized task during EEG recordings. For each participant, written consent was obtained before testing. The research was subject to ethical consideration by Nottingham Trent University’s Schools of Business, Law and Social Sciences Research Ethics Committee and has met with a favourable ethics opinion. It has been designed with reference to the British Psychological Society’s code of ethics.

### Depression

The Beck Depression Inventory II (BDI-II; Beck et al. 1996) consists of 21 items, assessing key elements of depression. Participants were instructed to rate each item on a scale from 0 to 3 according to symptom severity in the past two weeks. The total score was calculated as the sum of the items and can range from 0 (absence of depression) to 63 (severe depression). The BDI-II total has been validated in clinical and non-clinical populations (Wang and Gorenstein 2013) and found to have good internal consistency (Cronbach’s α = 0.90) and concurrent validity (Storch et al. 2004).

### Measurement of C-reactive protein (CRP)

Blood samples (10 mL) were obtained by antecubital venipuncture using BD Vacutainer^®^ Safety Lok™ blood collection sets containing ethylenediaminetetraacetic acid (EDTA) as an anticoagulant, and immediately placed on ice. Blood samples were centrifuged for 15 min (1000×g at 4 °C) within 30 min of collection. The plasma fraction was then aliquoted into cryotubes and stored at − 80 °C until analysis. ELISA was used to determine concentration of CRP (R&D Systems Quantikine^®^ ELISA) in 100-fold diluted plasma samples. These were assayed with a 96-well solid-phase quantitative sandwich enzyme immunoassay. Intra- and inter-assay precision was less than 10% and mean minimum detectable level of the assay was 0.010 ng/mL. A calibrator diluent was used to make a two-fold dilution series and prepare a set of standards. After adding to each well 50 µl of sample or standard, both were assayed in duplicate, and, for each one, duplicate readings were then averaged, and the averaged zero standard optical density was subtracted. A standard curve was generated by reducing the data using computer software (www.elisaanalysis.com) to generate a four-parameter logistic (4-PL) curve fit. Concentrations read from the standard curve were multiplied by the dilution factor and optical density was determined within 30 min using a microplate reader set to 450 nm with wavelength correction of 570 nm.

### Faecal sample Preparation and microbial analysis

Fecotainer^®^ (www.fecotainer.eu/) kits were used to collect faecal samples that were aliquoted (1 g per participant) and frozen at − 20 °C within 2 h of voiding. Stool samples were thawed and re-suspended in phosphate-buffered saline (PBS; 0.01 M phosphate buffer, 0.0027 M potassium chloride and 0.137 M sodium chloride) and homogenised in a stomacher for 2 min at 460 paddle beats per minute. The resulting faecal slurry was vortexed with 3-mm glass beads (VWR) for 30 s before being centrifuged at 400×g for 2 min at room temperature. The supernatant (375 µL) was fixed in 4% (w: v) (1125 µL) paraformaldehyde for 4 h at 4 °C. To remove paraformaldehyde, samples were centrifuged at 13,000×*g* in 1 mL PBS for 5 min at room temperature; this washing step was repeated two more times, then samples were re-suspended in 150 µL PBS and stored in ethanol (1:1 by *v*:*v*) at − 20 °C for enumeration by fluorescence in situ hybridisation (FISH).

Flow-FISH, using fluorescently labelled 16 S rRNA–targeted oligonucleotide probes (Sigma-Aldrich, Steinheim, Germany) labelled at the 5′ end with fluorochrome Alexa^®^647, was used for bacterial enumeration of the faecal samples. 3 commonly used 16 S rRNA oligonucleotide probes targeting specific groups of bacteria were used. These determined numbers of *Clostridium perfringens-histolyticum* group (Chis150;Franks et al. 1998) and *Lactobacillus-Enterococcus* (Lab158; Harmsen et al. 1999). The Eub338 mix probe (Daims et al. 1999; linked to the fluorochrome Alexa^®^488) was used to measure total bacteria within the samples via a fluorescence detector. The probe NonEub was used to control for non-specific Eub338 binding (Wallner et al. 1993). Bacterial groups of interest were assessed using probes (linked to fluorochrome Alexa^®^647) to fluoresce at a different excitation wavelength to be detectable in a fluorescence detector (FL4-H). Fixed samples were treated with lysozyme TE-FISH buffer (0.1 M Tris, 0.05 M EDTA, 1 mg/mL lysozyme) for 10 min at room temperature. The sample was washed twice in PBS and re-suspended in hybridization buffer (0.9 M NaCl, 0.02 M Tris/HCl, 30% formamide, 0.01% SDS), together with the oligonucleotide probe of interest. 50 µL of the hybridising mixtures with 4 µL of the corresponding probes were incubated overnight at 35 °C before 150 µL of hybridisation buffer was added to each tube, mixed, and centrifuged 3 min at 13,000×g. All Supernatant was removed and discarded using a pipette. 200 µL of washing buffer containing EDTA were added, and samples were incubated for 20 min at 37 C to remove nonspecific bonds between the DNA and the probes. The sample was again centrifuged for 3 min 13,000×*g* and the supernatant removed. The pellet was re-suspended in PBS solution and the sample was read using the Accuri C6 (BD Biosciences, Oxford, UK) flow cytometer.

### Experimental task

Participants performed a *Faces and Feedback* Task, in which they were instructed to observe emotional faces, presented one at a time. Participants were instructed to try to discover a secret rule contingent on the face stimuli presented. In doing so, they were asked to select one number from 1 to 5 after each face presentation. A feedback stimulus was then displayed, either a green tick (positive feedback) or a red cross (negative feedback). Participants were told to use the feedback to discover the secret rule. In actual fact, the secret rule was that the feedback was presented randomly. In total, each participant was presented with 50% positive and 50% negative feedback. Emotional faces (*n* = 200) were selected from an online database (http://www.macbrain.org/resources.htm). Stimuli consisted of 100 female and 100 male faces, presenting a total of 40 (20 male, 20 female) faces for each of five possible emotional expressions (anger, sadness, fear, happiness or neutral). For each trial, a fixation cross was presented in the centre of the screen for 500 ms. Subsequently, one emotional face was randomly presented in the centre of the screen for 700 ms, followed by an interstimulus interval (ISI 300 ms +/- 100 ms) and presentation of a question mark in the centre of the screen (“?”). Whilst the question mark was on screen, participants had to select one number from 1 to 5. Following button press, there was a further ISI (400 ms +/- 100 ms) prior to feedback stimulus which was presented for 500ms. Intertrial interval duration was 400 ms +/- 100 ms. The current study focuses on the response to feedback stimuli.

### EEG recording, preprocessing and erps extraction

Continuous EEG was recorded using a BioSemi Active II system (Biosemi, Amsterdam, The Netherlands). Recordings were taken from 64 active scalp electrodes based on the 10/20 system, at 2048 Hz and digitized at 24 bits. Data were referenced online with a CMS/DRL feedback loop. EEGLAB (Delorme and Makeig 2004) and MATLAB (Mathworks, Natick, Massachusetts, USA) were used for off-line analyses. Data were downsampled to 256 Hz and processed through a 1 Hz high pass filter and a 35 Hz low-pass filter. Bad channels were removed and interpolated. Data were then re-referenced to the linked mastoids. Epochs of 1200 ms (200 ms baseline before feedback presentation and 1000 ms after) were extracted. Independent component analysis (ICA) was used to remove ocular artifacts. Epochs containing remaining artifacts were rejected through visual inspection. Following baseline correction with respect to 200 ms before feedback presentation, stimulus-locked ERPs were averaged separately for each type of stimulus (positive and negative feedback). The FRN was quantified at Fz, Fcz, Cz, and Cpz, and Pz, by calculating the peak-to-peak amplitude, as difference between the negative peak in the 250–350 ms interval and the preceding positivity (Hajcak et al. 2006; Bellebaum et al. 2010).

### Statistical analyses

All analyses were performed using R (R Core Team 2020). A 2 (feedback type: positive, negative) x 5 (channel: Fz, FCz, Cz, CPz, Pz) repeated measures ANOVA was used to examine differences in FRN between positive and negative feedback across midline electrodes. Pearson’s correlations were used to explore whether FRN was associated with *Lactobacillus - Enterococcus*, *Clostridium histolyticum* group, depression, and CRP. Benjamini- Hochberg procedure (Benjamini and Hochberg 1995) with a false discovery rate criterion of 15% was used for multiple comparisons correction. Data were analysed using two level cross-classified multilevel models (MLM), because for each participant the FRN was quantified for positive and negative feedback. The dependent variable was FRN amplitude. Models included a random slope by feedback type, and two fully crossed random factors (subject and channel), estimated using an unstructured variance-covariance matrix. Feedback type was effect coded (negative = −0.5, positive = 0.5). In order to test the effect of microbiota on the FRN, *Lactobacillus - Enterococcus* and *Clostridium histolyticum* group were entered as predictors in separate models. Additionally, plasma CRP concentration, and depression were entered as predictors in both models. Cross-level interactions between microbiota and feedback type were included to assess whether microbiota moderated the effect of feedback type on FRN. *Lactobacillus - Enterococcus*,* Clostridium histolyticum*, CRP, and depression were grand-mean centered (Enders and Tofighi 2007). Satterthwaite corrections were used to estimate degrees of freedom and p-values. To fit the models, lme4 (Bates et al. 2014) and lmerTest (Kuznetsova et al. 2017) packages were used.

## Results

**Fig. 1:**
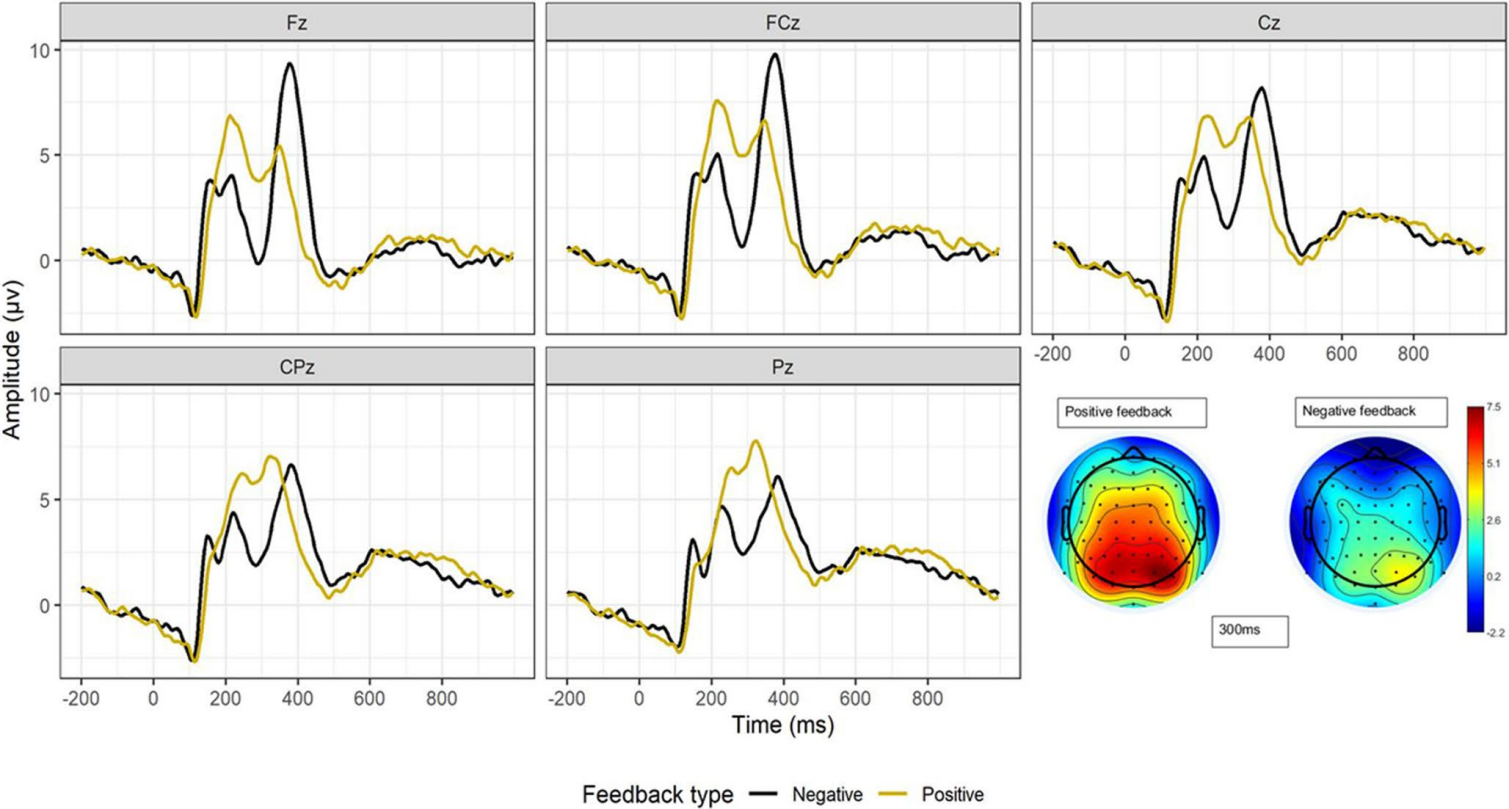
Feedback-locked grand-average waveforms for positive and negative feedback at channels Fz, FCz, Cz, CPz, and Pz and topographical plots of FRN at 300 ms

Descriptive statistics are displayed in Table 1. Grand averaged waveforms are shown in Fig. 1. A two-way repeated measures ANOVA was conducted to examine the effect of feedback type and channel on FRN amplitude. There was a main effect of feedback type, *F*_1,28_= 15.16, *p* <.001, and a main effect of channel, *F*_4,112_= 32.39, *p* <.001, with FRN amplitude being larger for negative feedback at anterior sites (Fig. 1). The interaction between feedback type and channel was not statistically significant, *F*_4,112_= 0.112, *p* =.978.

**Table 1:** Descriptive statistics, mean (SD) or N (%)

Age (years)	36.4 (11.8)				
Sex (m: f)	14:15 (48:52%)				
Depression (BDI-II)	5.8 (4.4)				
CRP (ng/ml)	11.7 (16.7)				
*Lactobacillus-Enterococcus*	7.5 (0.3)				
*Clostridium histolyticum* group	7.0 (0.4)				
**ERPs**	Fz	FCz	Cz	CPz	Pz
FRNn (µv)	−9.06 (6.17)	−8.72 (5.97)	−7.27 (5.20)	−5.94 (4.56)	−5.45 (4.04)
FRNp(µv)	−6.30 (3.78)	−5.78 (3.74)	−4.62 (3.58)	−3.16 (2.87)	−2.57 (2.37)

CRP, C-reactive protein; ERPs, event-related potentials, FRNn, feedback-related negativity for negative feedback; FRNp, feedback-related negativity for positive feedback. *Lactobacillus - Enterococcus* and *Clostridium histolyticum* are expressed as mean log_10_ cells/g fresh faeces

Correlational analyses revealed that FRN for positive feedback was associated with *Lactobacillus - Enterococcus* at FCz, *r*(27) = − 0.39, *p* =.038, Cz, *r*(27) = − 0.44, *p* =.018, CPz, *r*(27) = − 0.48, *p* =.008, and Pz, *r*(27) = − 0.45, *p* =.015, and with *Clostridium histolyticum* group at Cz, *r*(27) = − 0.37, *p* =.049, CPz, *r*(27) = − 0.41, *p* =.027, and Pz, *r*(27) = − 0.47, *p* =.011. Given that FRN is measured as a negative deflection, results indicate that larger FRN amplitude for positive feedback is associated with greater numbers of microbiota. No statistically significant correlations between FRN, CRP, and depression were found.

MLM analyses are summarized in Table 2. For the intercept-only model, the mean intercept, *b*=−5.88, 95% CI [−7.78, −3.99], *t*(11.75)=−6.08, *p* <.001, was significant. Between-individual variability in the FRN accounted for 51% of the total variance and variance between channels accounted for 10% of the total variance.

**Table 2:** Multilevel models testing the effect of microbiota, CRP, and depression on FRN amplitude. AIC, Akaike information criterion; ICC, intraclass correlation coefficient; CRP, C-reactive protein

Intercept-only	B	CI	df	t	p
(Intercept)	−5.88	−7.78, −3.99	11.75	−6.08	<.001
Random effects	Variance	SD	ICC		
subject	12.11	3.48	.51		
channel	2.44	1.56	.10		
Residual	9.03	3.01			
Model 1^a^	B	CI	df	t	p
(Intercept)	−5.89	−7.75, −4.02	10.93	−6.22	<.001
Feedback type	2.80	1.36, 4.24	27	3.82	<.001
*Clostridium histolyticum* group	−3.44	−6.67, −0.20	27.15	−2.09	<.05
CRP	−0.07	−0.13, −0.01	25	−2.08	<.05
Depression	−0.08	−0.31, 0.16	25	−0.65	.525
Feedback type x *Clostridium histolyticum* group	−0.23	−3.83, 3.36	27	−0.13	.899
Model 2^b^	B	CI	df	t	p
(Intercept)	−5.89	−7.77, −4.00	11.35	−6.15	<.001
Feedback type	2.80	1.37, 4.23	27	3.86	<.001
*Lactobacillus*—*Enterococcus*	−2.85	−6.94, 0.61	25.88	−1.65	.111
CRP	−0.04	−0.11, 0.02	25	−1.39	.176
Depression	−0.08	−0.31, 0.16	25	−0.6	.524
Feedback type x *Lactobacillus*—*Enterococcus*	−1.56	−5.28, 2.70	27	−0.72	.477

^a^ AIC of the main effect model was 1347.01; inclusion of the interaction resulted in AIC of 1348.47

^b^ AIC of the main effect model was 1349.41; inclusion of the interaction resulted in AIC of 1347.51

There was a main effect of feedback type on FRN amplitude, *b* = 2.80, 95% CI [1.36, 4.24], *t*(27) = 3.82, *p* <.001, for both models, showing that FRN amplitude for positive feedback was lower (more positive) as compared to negative feedback.

Furthermore, in model 1, there was a significant main effect of *Clostridium histolyticum* group, *b*= −3.44, 95% CI [−6.67, −0.20], *t*(27.15)=−2.09, *p* <.05, indicating that higher levels of clostridia group I and II were associated with larger FRN amplitude. There was a main effect of CRP, *b*=−0.07, 95% [−0.13, −0.01], *t*(25)=−2.08, *p* = < 0.05, showing that higher inflammation was associated with larger FRN amplitude. The main effect of depression and the interaction of Feedback type and clostridia were not significant.

In model 2, the main effects of lactobacilli, CRP, or lactobacilli, and the interaction between lactobacilli and feedback type were not significant.

## Discussion

Using a novel *faces and feedback* ERP task, the current study is the first to investigate the association between gut microbiota and FRN. In doing so, it contributes important empirical information regarding mechanisms linking the gut and brain. Correlation analysis suggested that higher FRN amplitudes in response to positive feedback were associated with greater numbers of *Lactobacillus - Enterococcus* and *Clostridium histolyticum* group. In the MLM, however, FRN amplitude was found to be associated with *Clostridium histolyticum*, but not *Lactobacillus - Enterococcus*, abundance after controlling for inflammation and depression. These findings suggest that specific microbial groups are associated with neurophysiological mechanisms underlying cognitive functioning, in line with previous evidence suggesting a relation between gut microbiota and frontal functional network connectivity (Wang et al. 2019; Cai et al. 2021) and task-related brain activity (Callaghan et al. 2020). Our findings are in line with a relationship between ANS activity and FRN (Kimura 2019), implicating the contribution of gut-brain communication to the emergence of feedback processing ERPs. Alternatively, however, because of the cross-sectional design in the current study, findings might equally reflect an effect of self-monitoring mechanisms underlying FRN generation on gut microbiome, for example by predisposing to certain temperaments and psychological states (e.g., stress responsivity; Sumich et al., 2022). 

Within the *Clostridium histolyticum* group, there is also the possibility to detect *Clostridium butyricum.* Neuroprotective properties of *C. butyricum* treatments have been widely reported. Although it remains unclear about the molecular mechanisms underlying their CNS actions, it has been suggested that changes occurring in the brain following gut microbiota modulation may be related to increased diversity of gut microbiota. For example, *C. butyricum* treatments could induce significant rise of butyrate content in both the gut and the brain (Liu et al. 2015). Butyrate is a short-chain fatty acid, which not only acts as an energy source for intestinal epithelial cells, but also has effects on anti-inflammatory properties via its inhibition of histone deacetylase (HDAC; Vinolo et al. 2011) and on dopamine regulation (Hamamah et al. 2022). FRN has been related to risk-taking and reinforcement learning proficiency and considered to reflect underlying individual differences in midbrain dopamine functioning. Indeed, several studies showed that dopamine function moderates FRN amplitude (Walsh and Anderson 2012; Webber et al. 2021). Therefore, future studies should further investigate the dynamic interaction between microbiota and dopamine level in reward-related learning and performance and explore whether phasic/tonic dopaminergic release may be associated with abundance *Clostridium* spp in healthy individuals.

It has been previously proposed that “gut feelings” play an important role in decision-making (Mayer 2011) and executive function (Roman et al. 2018; Cai et al. 2021). Gut signals modulated by microbiota composition may contribute to ANS signals integrated by anterior insula and medial prefrontal cortex (Mayer 2011), for example via vagus nerve communication (Bravo et al. 2011), to trigger behavioural adjustments in order to improve performance (Ullsperger et al. 2014) following feedback processing. Interestingly, previous research explored the role of peripheral preparatory inputs related to error awareness, that in ERP research is typically studied in relation to response-locked ERPs, i.e., error-related negativity and error positivity (Hajcak et al. 2003; O’Connell et al. 2007; Wessel et al. 2011; Maier et al. 2019), when no feedback is presented, and post-error behavioural adjustments mainly rely on the internal evaluation of action outcomes. According to the *Accumulation account* (Ullsperger et al. 2010; Wessel et al. 2011), performance monitoring processes following conscious perception of error rely on the integration of peripheral signals mediated by posterior mesial frontal cortex and insular activity (Klein et al. 2013; Ullsperger et al. 2014). It could be argued that similar processes may be activated by feedback presentation when external evaluation of performance leads to awareness of response correctness. The association between FRN and microbiota was found to be stronger following positive feedback when compared to negative feedback. For many years the FRN has been considered as a “negativity”, presenting larger amplitude following negative feedback as compared to positive feedback (Krigolson 2018). However, previous research referred to the FRN as reward positivity (RwP; Proudfit 2015), based on evidence suggesting that feedback processing may modulate the positive, but not the negative conditional waveform (Holroyd et al. 2008; Krigolson et al. 2014). Moreover, it has been suggested that positive feedback may be more relevant for the implementation of neurobehavioral adjustments and learning (Zioga et al. 2019).

In the current study, FRN amplitude was not associated with self-reported depressive symptoms. These findings, therefore, do not support the hypothesis of reduced FRN as a biomarker of depression (Proudfit 2015), which is indeed still debated (Clayson et al. 2020). The association between performance monitoring ERPs and depression has been found to be stronger in clinical populations and inconsistent findings may be explained by other factors, such as experimental manipulations (Weinberg et al. 2015; Clayson et al. 2020). Crucially, our analysis revealed that in model 1 there was a main effect of inflammation, which has been proposed to play a pivotal role in the development of depression (Miller and Raison 2016; Beurel et al. 2020). Therefore, it could be hypothesized that bidirectional BGMA communication, including inflammatory mechanisms may mediate the relationship between depressed mood and reward processing (Dooley et al. 2018). Moreover, the current study used BDI total scores, which combine cognitive and non-cognitive aspects of depressive symptoms. Some evidence suggests that the relation between feedback-related ERPs and depression may be specific to certain depressive symptoms (e.g., Mueller et al. 2015). Future studies should incorporate psychometric assessments that precisely target different facets of depression.

MLM revealed that FRN was associated with *Clostridium histolyticum* group, but not with *Lactobacillus - Enterococcus*, suggesting the existence of relationships between specific genera of microbiota and neurophysiological correlates of performance monitoring. Similarly, previous ERP research showed that N1, N2, and P300 were associated with specific phyla and with microbial diversity (Canipe et al. 2021). This is also supported by further fMRI evidence (Callaghan et al. 2020), showing that task-related brain activation clusters presented associations with certain genera (*Bacteroides* and *Lachnospiraceae).* However, it is important to note that the present study focused on a limited number of genera. Future studies should explore the association between performance monitoring ERPs and microbiota at different taxonomic levels, and include measures of microbial diversity, such as alpha (within-subject) and beta (between-subject) diversity.

Considering the moderate associations between FRN and microbiota revealed by the current study, further research is needed to extend our knowledge about the role of BGMA in self-monitoring processes, and specifically the interaction between inflammation, depression, and reward-related ERPs. Importantly, the current task included zero-value random feedback presentation (ticks or crosses). Future investigations should employ learning-based tasks to understand the relationship between gut-brain interactions and neurobehavioral adjustments; and single-trail analysis to explore intra-individual FRN trial-level changes (Volpert-Esmond et al. 2018). Varying values of feedback (e.g., monetary wins/losses) could be used to elucidate the mediating effect of feedback magnitude.

The current study has important implications for understanding the role of the gut microbiota in neurocognitive function in healthy individuals and for the therapeutic use of prebiotics and probiotics in clinical settings. Over the last two decades, an exponential increase in research in gut microbiota has led to greater understanding of their role in the pathophysiology of clinical conditions. For example, atypical diversity and microbial dysbiosis (or dysbacteriosis) have been implicated in psychiatric and neurological disorders (e.g., Pistollato et al. 2016; Simpson et al. 2021; Sun and Shen 2018; Vogt et al. 2017; Yuan et al. 2019). This has paralleled development of theories around the involvement of the immune system and inflammation in these psychological noncommunicable diseases (Grochowska et al. 2019; Suganya and Koo 2020). Emerging evidence indicates benefits from prebiotic and probiotic supplementation on cognition (Mörkl et al. 2020; Paiva et al. 2020; Lv et al. 2021; Kang and Zivkovic 2021), which present additional intervention advantages, such as easy implementation through dietary changes (Paiva et al. 2020) and lack of addictive properties contained in pharmacological medications that may cause adverse side-effects (Liu 2017). However, neural changes associated with therapeutic effects, remain relatively uninvestigated (Paiva et al. 2020). Performance monitoring ERPs may offer a target for gut microbiota-based interventions to assess neurocognitive benefits.

In conclusion, the present study is the first to investigate the relationship between neurophysiological processes underlying self-monitoring and BGMA function. As such, these findings offer new perspectives on self-monitoring ERPs as a possible target to evaluate novel BGMA interventions on cognition and behaviour in healthy individual and clinical populations.

## Supplementary Information

Below is the link to the electronic supplementary material.

Supplementary Material 1

## Data Availability

Data requests are considered by the authors, and requests should be made via email.
