# Supplementary material for: Investigating the relationship between gut microbiota and electrocortical signatures of feedback processing: an ERP study
Source: Psychopharmacology (Berl). 2025 Aug 29;243(4):767–78. doi: 10.1007/s00213-025-06878-9 (PMC13035655; doi:10.1007/s00213-025-06878-9)
Supplement: Supplementary file 1 — Supplementary Material 1 [file 213_2025_6878_MOESM1_ESM.docx]

Supplementary Analyses inlcuding ΔFRN.

ΔFRN was computed as difference between positive and negative waform. Mean amplitude was computed in the 250-350ms interval following feedback presentation.

**Descriptive**

| ΔFRN (Fz) | ΔFRN (FCz) | ΔFRN (Cz) | ΔFRN (CPz) | ΔFRN (Pz) |
| --- | --- | --- | --- | --- |
| 2.49 (2.36) | 2.68 (2.31) | 2.77(2.28) | 3.37(2.38) | 2.64 (2.53) |

**Correlations**

ΔFRN *(Fz)* positively correlated with LAB (r=.0373, p=.037). No other statically signifcant correlation between ΔFRN and other variables was found.

**Mulitlevel models**

|  | ΔFRN | | |
| --- | --- | --- | --- |
| *Predictors* | *Estimates* | *CI* | *p* |
| (Intercept) | 2.99 | 2.11 – 3.87 | **<0.001** |
| CHIS | -0.28 | -2.41 – 1.85 | 0.796 |
| CRP Con | -0.01 | -0.06 – 0.04 | 0.629 |
| BDI total | -0.07 | -0.25 – 0.12 | 0.477 |
| **Random Effects** | | | |
| σ^2^ | 0.95 | | |
| τ_00_ _subject_ | 4.42 | | |
| τ_00_ _site_ | 0.20 | | |
| ICC | 0.83 | | |
| N _subject_ | 29 | | |
| N _site_ | 5 | | |
| Observations | 145 | | |
| Marginal R^2^ / Conditional R^2^ | 0.024 / 0.833 | | |

|  | ΔFRN | | |
| --- | --- | --- | --- |
| *Predictors* | *Estimates* | *CI* | *p* |
| (Intercept) | 2.99 | 2.07 – 3.91 | **<0.001** |
| LAB | -1.28 | -3.78 – 1.22 | 0.315 |
| CRP Con | -0.01 | -0.06 – 0.04 | 0.644 |
| BDI total | -0.06 | -0.25 – 0.13 | 0.554 |
| **Random Effects** | | | |
| σ^2^ | 0.95 | | |
| τ_00_ _subject_ | 4.92 | | |
| τ_00_ _site_ | 0.21 | | |
| ICC | 0.84 | | |
| N _subject_ | 29 | | |
| N _site_ | 5 | | |
| Observations | 145 | | |
| Marginal R^2^ / Conditional R^2^ | 0.048 / 0.851 | | |
